# Exercise Training-Induced MicroRNA Alterations with Protective Effects in Cardiovascular Diseases

**DOI:** 10.31083/j.rcm2409251

**Published:** 2023-09-06

**Authors:** Juan Gao, Jiaxin Song, Yuwei Yan, Priyanka Gokulnath, Gururaja Vulugundam, Guoping Li, Qingyi Zhan, Fei Jiang, Yanjuan Lin, Junjie Xiao

**Affiliations:** ^1^Institute of Geriatrics (Shanghai University), Affiliated Nantong Hospital of Shanghai University (The Sixth People's Hospital of Nantong), School of Medicine, Shanghai University, 226011 Nantong, Jiangsu, China; ^2^Cardiac Regeneration and Ageing Lab, Institute of Cardiovascular Sciences, Shanghai Engineering Research Center of Organ Repair, School of Life Science, Shanghai University, 200444 Shanghai, China; ^3^Cardiovascular Division of the Massachusetts General Hospital and Harvard Medical School, Boston, MA 02114, USA; ^4^Biologics Development, Sanofi, Framingham, MA 01701, USA; ^5^Department of Nursing, Union Hospital, Fujian Medical University Union Hospital, 350001 Fuzhou, Fujian, China; ^6^Fujian Provincial Special Reserve Talents Laboratory, Fujian Medical University Union Hospital, 350001 Fuzhou, Fujian, China

**Keywords:** exercise training, beneficial adaption, cardiovascular diseases, microRNA, therapeutic targets

## Abstract

Exercise training (ET) is an important non-drug adjuvant therapy against many human diseases, including cardiovascular diseases. The appropriate ET intensity induces beneficial adaptions and improves physiological function and cardiopulmonary fitness. The mechanisms of exercise-induced cardioprotective effects are still not fully understood. However, mounting evidence suggest that microRNAs (miRNAs) play crucial role in this process and are essential in responding to exercise-stress and mediating exercise-protective effects. Thus, this review summarizes the biogenesis of miRNAs, the mechanism of miRNA action, and specifically the miRNAs involved in exercise-induced cardio-protection used as therapeutic targets for treating cardiovascular diseases.

## 1. Introduction

MicroRNAs (miRNAs) are small, single-stranded, evolutionally conserved, non-coding RNAs composed of 19 to 24 nucleotides (nt). The first miRNA, lin-4, was discovered in 1993 in *Caenorhabditis elegans* and is essential in regulating postembryonic developmental processes [1]. Since then, numerous miRNAs have been identified in different types of organisms with diverse functions substantially elucidated [2, 3, 4]. Currently, more than 2000 miRNAs have been discovered in humans, which regulate about one-third of the protein-coding genes. miRNAs are closely associated with many diseases and are being explored as novel diagnostic and therapeutic strategies [5].

miRNAs recognize and bind to their target mRNAs via base-paring and exert their activity either by inhibiting mRNA translation or by promoting messenger RNA (mRNA) decay at the post-transcriptional level. miRNAs are involved in many fundamental biological processes based on cell-signaling, such as cell proliferation, cell growth, cell metabolism, cell morphogenesis, and apoptosis. The function of an individual miRNA has been understood by miRNA silencing or overexpression *in vitro* or *in vivo* [6]. Dysregulation of miRNAs leads to development of various diseases, including cardiovascular diseases, nervous system diseases, cancer, and infectious diseases [7, 8].

Exercise-training (ET) causes physical stress and affects the body in different ways. Muscle tissues, cardiopulmonary systems, and multiple organs respond to the exercise stimulus. Appropriate exercise stress induces beneficial changes in the whole body, improves tissue metabolism, and increases oxidative capacity as well as cardiopulmonary fitness [9, 10]. Various response factors mediate the adaptive changes induced by exercise, and miRNAs are one of the crucial executors [11, 12].

Here, we elucidate the biogenesis of miRNAs along with their mechanism of action, emphasizing on miRNAs involved in exercise-induced cardio-protection, especially because they could be used as therapeutic targets for cardiovascular diseases.

## 2. The Biogenesis and Function of miRNAs

**Fig. 1.**
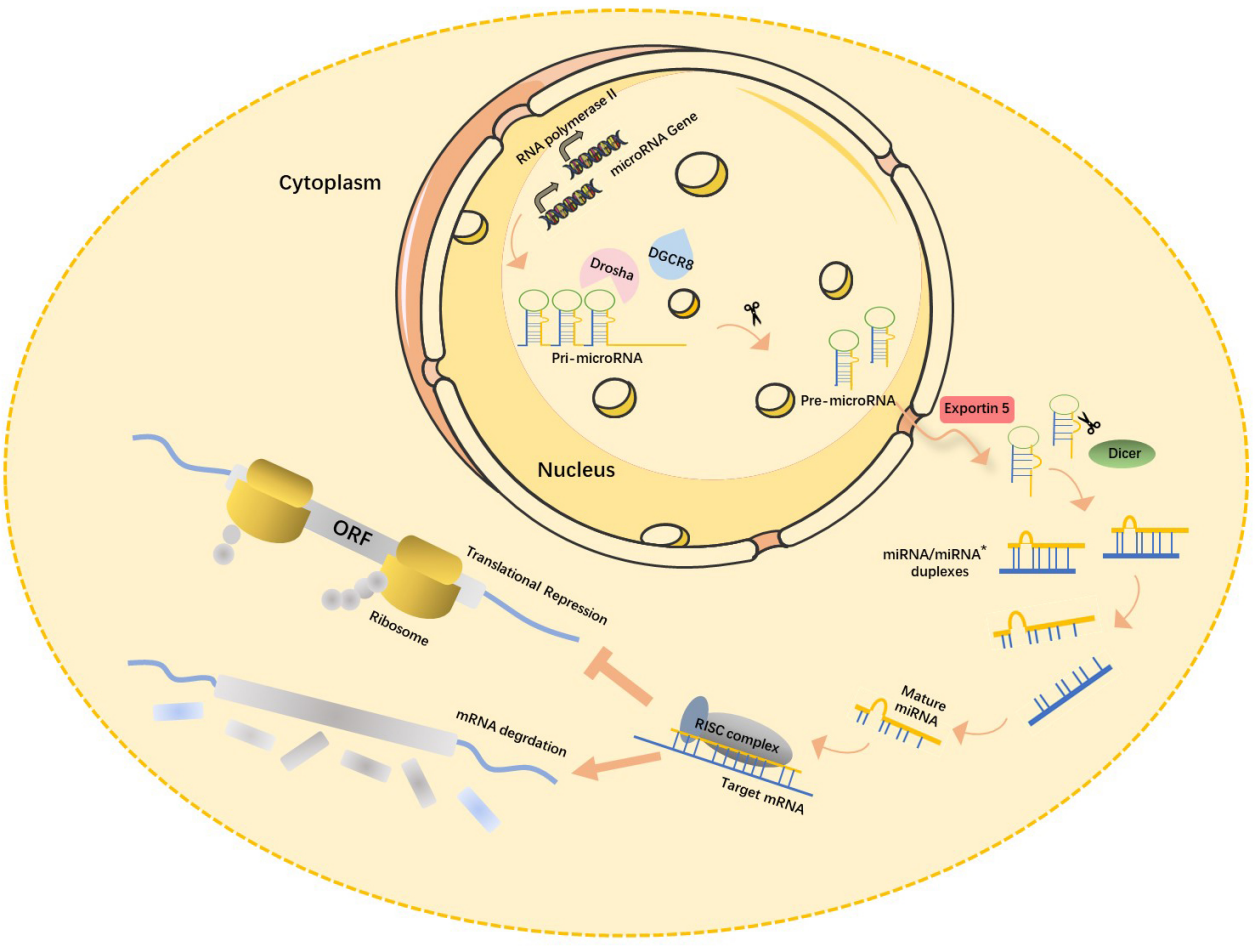
**Canonical miRNAs biogenesis pathway**. In the nucleus, pri-miRNA, the primary transcription product of the miRNA gene, is cleaved by RNase III-Drosha enzyme to become hairpin precursor miRNA (pre-miRNA). After preliminary shearing, the pre-miRNA is transported from the nucleus to the cytoplasm under the action of the transporter Esportin-5, and then further cleaved by another RNase III Dicer enzyme to produce mature miRNA. The mature miRNAs then bind with other proteins to form RISC (RNA-induced silencing complex), which leads to target mRNA degradation or translation inhibition. miRNA, microRNAs; pri-miRNA, primary miRNA transcripts; DGCR8, DiGeorge syndrome critical region 8; ORF, open reading frame; mRNA, messenger ribonucleic acid; miRNA*, star strand of microRNA.

Recent studies have shown that about 70% of mammalian miRNA genes are located in the transcription units (TUs) regions, mostly in their intronic region. miRNA-encoding genes located in the intron region are usually highly conserved among different species both in their genome locus and sequence homology of mature miRNA [13], which in turn is closely associated with their functional importance. Most miRNA-encoding genes exist either as a single copy, multiple copies, or clusters in the genome [14]. The biogenesis of miRNAs is under strict temporal and spatial transcriptional control, resulting in a high diversity of their expression profile. miRNA encoding genes are generally transcribed by RNA polymerase II in the nucleus, and the length of the primary miRNA transcripts (pri-miRNA) can be more than 1000 nucleotides. Canonical miRNAs are processed in multiple steps (Fig. 1) [15]. First, double-stranded RNA-specific endoribonuclease (DROSHA) forms a microprocessor complex with DiGeorge syndrome critical region 8 (DGCR8). It then recognizes the specific hairpin structure and the length of pri-miRNA and cleaves it into miRNA precursors (pre-miRNA, normally 60–70 nt) using its ribonuclease enzyme activity. Next, the Exportin5 (Exp5)/RanGTP mediates the nuclear export of pre-miRNA by forming a pre-miRNA/Exp5/RanGTP complex. Then, Dicer, a Ribonuclease III (RNase III) endonuclease, cleaves the hairpin miRNA precursors into mature miRNA duplex (miRNA:miRNA*), a form of short double-stranded RNA (dsRNA) [16].

While mature miRNA, also called the guide or leading strand, was considered the biologically active miRNA, the miRNA star (miRNA*)/passenger strand/carrier strand, was formerly thought to be inactive. Currently, the miRNA duplex is named according to its position in the pre-miRNA hairpin structure. While the miRNA-5p strand is located in the forward ($5^{\prime }$${\mathrm{-3}}^{\prime }$) position, its nearly complementary strand, the miRNA-3p, is presented in the reverse position ($3^{\prime }$${\mathrm{-5}}^{\prime }$). Certain studies in recent years have shown that both miRNA-5p and miRNA-3p strands are functional in specific developmental stages or species [17]. Mature miR-17-5p can coordinate with its passenger strand miR-17-3p to target tissue inhibitor of metallopeptidase 3 (*TIMP3*), leading to increased tumor cell proliferation, survival, and invasion, ultimately inducing prostate tumor growth and invasion [18]. Depletion of miR-21 could abrogate circadian regulation of apoptosis and reduce necrotic core size in atherosclerotic lesions. The effect of each miR-21 strand, miR-21-5p and miR-21-3p in this process were characterized, and both strands were identified to target different $3^{\prime }$*-*untranslated regions (UTR) of XIAP-associated factor 1 (*XAF1*) through noncanonical target sites and inhibit macrophage apoptosis [19]. However, not all miRNAs have this feature of contribution by both strands. Depletion of miR-126 has been shown to impair endothelial recovery after mechanical injury. A mechanistic study clarified that endothelial miR-126-5p, but not the passenger strand miR-126-3p could promote endothelial proliferation and inhibit atherosclerotic lesion formation by targeting the NOTCH1 inhibitor delta-like 1 homolog (*DLK1*) [20]. Another study found that miRNA-126-5p and miRNA-126-3p had different expression profiles and subcellular localization in rapamycin-administrated endothelial cells, indicating that they were modulated by different post-transcriptional strand regulation mechanisms [21]. Analysis of miRNAs in cardiac fibroblast–derived exosomes revealed that they were enriched with passenger strands miRNA-3p. Further study found that miR-21-3p in exosomes could be released from cardiac fibroblasts and transferred to cardiomyocytes, acting as a paracrine signaling mediator and leading to cardiomyocyte hypertrophy [22].

One strand of miRNA (either -5p or -3p) is incorporated into an RNA-induced silencing complex (RISC), a multi-protein large molecular weight complex. It is used as a template to recognize and bind to its target mRNA by base-paring, leading to mRNA degradation or translation inhibition through different mechanisms [23]. In plants, most miRNAs bind to their target mRNAs by exact or nearly exact complementary base-pairing. However, most miRNAs and their mRNA targets in animals have imperfect complements and fewer sequence homologies. The miRNA binding sites are located in all the regions of mRNAs, including coding sequence, $5^{\prime }$UTR, and $3^{\prime }$UTR, but vary in their relative proportions. A technique for ligation and sequencing of human AGO1-associated miRNA-target RNA duplexes, named crosslinking, ligation, and sequencing of hybrids (CLASH), was developed, to obtain an unbiased view of miRNA-target interactome and to reevaluate the rules of miRNA-mRNA binding sites. This study found that 60% of binding sites of all miRNA were revealed in the coding sequence of mRNA targets, 35% were in the $3^{\prime }$UTR regions, and 5% were mapped to the $5^{\prime }$UTR [24]. Thus, the mechanism of miRNAs in regulating target mRNA has high flexibility in animals. Moreover, one miRNA can bind to multiple target mRNAs, and different miRNAs can target the same target mRNA, which adds to the complexities of the miRNA signaling [25].

The function of the RISC complex is mainly mediated by the argonaute (AGO) protein together with multiple associated proteins, and the miRNA function is primarily due to its incorporation into the RISC complex. Four AGO proteins (AGO1–4) were encoded in the mammalian genome, and AGO2 is the most highly expressed and the only AGO protein that retains the nuclease activity to cleave miRNA targets in humans [26]. AGO proteins can determine the mechanism by which RISC plays a role in gene regulation. In the cytoplasm, miRNAs can recognize and bind to their mRNA targets. Further, they can also be released from their former mRNA targets and rebind to different mRNA targets, thereby continuously modulating many target molecules. Emerging evidence shows that miRNAs not only regulate genes at the post-transcriptional level resulting in transcriptional gene silencing (TGS), but also mediate transcriptional gene activation (TGA) in the nucleus, acting at a transcriptional level [27]. In addition to regulating the mRNA decay or mRNA translation in the cytoplasm, miRNAs could affect gene transcription and expression in the nucleus by altering the epigenetic status of gene promoters and enhancers, and by regulating gene-derived transcripts in the mitochondrion [28]. miRNAs hybridize with double-stranded DNA and bind specifically to the promoter region of a gene, therefore regulating gene transcription. miRNAs can inhibit the maturation of noncoding RNA by interacting with them through complementary sequences. miRNAs affect alternative splicing through mediators such as AGO proteins. In the nucleolus, miRNAs also regulate the stability of mRNA and ribosomal ribonucleic acid (rRNA) [29]. In addition, miRNAs can interact directly with proteins and affect their functions, thus exerting crucial impact on cardiovascular biology. Endothelial miR-126-5p was found to bind to *caspase-3* (*CASP3*), to suppress caspase dimerization and inhibit its activity thus reducing cell apoptosis [21]. miR-1-3p could bind to cardiac membrane protein, such as inward-rectifier potassium channel *KIR2.1*, playing a critical role in the regulation of cardiac electrophysiology and arrhythmia [30].

Interestingly, miRNAs not only regulate the host cells from which they are generated. Mature miRNA duplexes could also be directly transferred to neighboring cells through gap junctions. Further, miRNAs can be secreted and transferred via exosomes or different types of small extracellular vesicles regulating corresponding mRNA targets in distal cells. In addition, high-density lipoprotein (HDL) has been shown to transport miRNAs into cells [7]. miRNAs also exist in serum and other body fluids termed circulating miRNAs, which can be used as risk factors or biomarkers for the diagnosis and prognosis of certain diseases [31]. Robust evidence has shown the predictive value of circulating miR-30d as a functionally validated RNA biomarker in acute heart failure (AHF) patients [19, 32, 33]. The sensitivity and specificity of miR-30d as a novel biomarker imply its promising role in a clinical setting. There is evidence of a prognostic role for miR-125b-5p in patients with cardiovascular disease. Stroke is currently the second most common cause of death worldwide and a leading cause of long-term disability. Computed tomography (CT) is normally used to diagnose hemorrhagic stroke in clinical settings. However, 40–50% of acute ischemic stroke (IS) cases showed no abnormality in admission CT scan. It was found that the expression levels of miR-125a-5p, miR-125b-5p and miR-143-3p were correlated with infarct size and stroke etiology. Area under the curve (AUC) of three miRNAs was 0.90 (sensitivity: 85.6%; specificity: 76.3%). This was far better than multiple previously reported biomarkers of acute IS, suggesting their potential use as widely useful diagnostic markers. Specifically, elevated levels of these three miRNAs indicate the early stages after stroke, and their peak expression could more accurately determine symptom onset [34]. In a screening for circulating miRNA with prognostic value for heart failure (HF) drug-refractory patients undergoing cardiac resynchronization therapy revealed that lower expression of miR-499a-5p and miR-125b-5p is closely associated with the improved left ventricular ejection fractions (LVEF). This provides evidence for their predictive potency [35]. Further studies on patients with both acute coronary syndrome (ACS) and multivessel disease (MVD) confirmed that those patients with plasma miR-125b-5p expression levels below 4.6 had better long-term all-cause survival [36]. Therefore, miRNAs can be secreted from donor cells and transferred to adjacent or remote recipient cells by different carriers, such as exosomes, microvesicles, and HDL, playing essential roles in intercellular communication [7].

## 3. Exercise-Responsive miRNAs in Cardiovascular Diseases

The role of miRNAs in cardiovascular physiology and pathology has been comprehensively studied [37]. The expression of a set of cardiac miRNAs in response to exercise with cardio-protective roles to regulate cell proliferation, metabolism, and apoptosis has been reported [38, 39, 40] (Table 1, Ref. [41, 42, 43, 44, 45, 46, 47, 48, 49, 50, 51, 52, 53, 54, 55, 56, 57, 58, 59, 60, 61, 62]). In response to physical exercise, these exercise-driven miRNAs have important roles in regulating exercise adaptation and could be used as promising therapeutic intervention. Moreover, exercise-regulated miRNAs can also be used as novel prognostic tools in many subareas of cardiology, such as HF [39, 40].

**Table 1.** **Exercise-responsive miRNAs, function and target genes**.

miRNA	Changes of miRNA after exercise	Targets	Functional effects	References
miR-126-3p	up	*SPRED1, PIK3R2*	Increased angiogenesis	[41, 42]
miR-21a-5p	up	*FABP7, HMGCR, ACAT1, OLR1*	Regulated lipid metabolism and improved hyperlipidemia	[43]
miR-214-3p	down	*SERCA2A*	Improved cardiac contractility and LV compliance	[44]
miRNA-1-5p	up	*NCX1*	Improved cardiac contractility and LV compliance	[44]
miR-133a-5p	up	*CASP3, CASP8, CASP9*	Reduced cardiac fibrosis and apoptosis	[45]
miR-1192	up	*CASP3*	Reduced cardiomyocyte apoptosis	[46]
miRNA-497-5p	down	*CLCN3, BCL-2*	Reduced cardiomyocyte apoptosis and inflammation	[47]
miR-29a-3p	up	*TGF-β1, SMAD2/3, COL1A1, COL3A1*	Reduced cardiac fibrosis	[48]
miR-101a-3p	up	*FOS, TGF-β1*	Reduced cardiac fibrosis	[48]
miR-29c-3p	up	*COL1A1, COL3A1*	Reduced cardiac fibrosis, improved LV compliance	[49]
miR-20a-5p	up	*PTEN*	Promoted the survival and proliferation of endothelial cells	[50]
miR-146a-5p	up	*TRAF6*	Reduced vascular inflammation injury	[51]
miR-125b-5p	up	*MAP3K5, MAP2K7, MAP2K4*	Reduced cardiomyocyte apoptosis	[52]
miR-128-3p	up	*MAP3K5, MAP2K7, MAP2K4*	Reduced cardiomyocyte apoptosis	[52]
miR-30d-5p	up	*MAP3K5, MAP2K7, MAP2K4*	Reduced cardiomyocyte apoptosis	[52]
miR-342-5p	up	*CASP9, JNK2, PPM1F*	Reduced cardiomyocyte apoptosis	[53]
miR-17-3p	up	*TIMP-3*	Promoted cardiomyocyte hypertrophy, proliferation, and survival	[54]
miR-222-3p	up	*HIPK1, HMBOX1, P27*	Promoted cardiomyocyte growth, proliferation, and survival	[55]
miR-486-5p	up	*PTEN, FOXO1, MST1 (STK4)*	Reduced cardiomyocyte apoptosis	[55]
miR-16-5p	down	*VEGF, BCL-2*	Increased angiogenesis	[56]
miR-344g-5p	up	*HMGCS2*	Reduced cardiomyocyte apoptosis	[57]
miR-455-5p	up	*MMP9*	Reduced cardiac fibrosis and myocyte uncoupling	[58]
miR-181b-5p	up	*PTEN, KPNA4*	Alleviated endothelial dysfunction and vascular inflammation	[59]
miRNA-208a-3p	down	*MED13, SOX6, SP3, PURβ, HP1β*	Induced physiological hypertrophy	[60]
miR-210-3p	down	*EFNA3*	Increased angiogenesis	[61]
miR-34a-5p	down	*SIRT1, CYCLIN D1, BCL-2*	Promoted cardiomyocyte proliferation and survival	[62]

Table notes: miRNA, microRNA; *SPRED1*, sprouty related EVH1 domain containing 1; *PIK3R2*, phosphoinositide-3-kinase regulatory subunit 2; *FABP7*, fatty acid binding protein 7; *HMGCR*, 3-hydroxy-3-methylglutaryl-CoA reductase; *ACAT1*, acetyl-CoA acetyltransferase 1; *OLR1*, oxidized low density lipoprotein receptor 1; *SERCA2A*, Sarco/endoplasmic reticulum Ca(2+)-ATPase; *NCX1*, Sodium/Calcium exchanger protein; *CASP3*, cysteinyl aspartate specific proteinase 3; *CASP8*, cysteinyl aspartate specific proteinase 8; *CASP9*, cysteinyl aspartate specific proteinase 9; *CLCN3*, chloride voltage-gated channel 3; *BCL-2*, B-cell lymphoma-2; *TGF-β1*, transforming growth factor Beta 1; *SMAD2/3*, SMAD family member 2/3; *FOS*, Fos proto-oncogene, AP-1 transcription factor subunit; *COL1A1*, collagen type I α1; *COL3A1*, type III collagen; *PTEN*, phosphatase and tensin homolog; *TRAF6*, TNF receptor associated factor 6; *MAP3K5*, mitogen-activated protein kinase kinase kinase 5; *MAP2K7*, mitogen-activated protein kinase kinase 7; *MAP2K4*, mitogen-activated protein kinase kinase 4; *JNK2*, c-Jun N-terminal kinase; *PPM1F*, protein phosphatase, ${\mathrm{Mg}}^{2+}$/${\mathrm{Mn}}^{2+}$ dependent 1F; *TIMP-3*, tissue inhibitor of metalloproteinase 3; *HIPK1*, homeodomain interacting protein kinase 1; *HMBOX1*, homeobox containing 1; *P27*, cyclin-dependent kinase inhibitor 1B; *FOXO1*, forkhead box O1; *MST1*, macrophage stimulating 1; *STK4*, serine/threonine kinase 4; *VEGF*, vascular endothelial growth factor ; *HMGCS2*, 3-hydroxy-3-methylglutaryl-CoA synthase 2; *MMP9*, matrix metallopeptidase 9; *KPNA4*, karyopherin subunit alpha 4; *MED13*, mediator complex subunit 13; *SOX6*, SRY-box transcription factor 6; *SP3*, Sp3 transcription factor; *PURβ*, purine rich element binding protein Beta; *HP1β*, Heterochromatin Protein 1 Beta; *EFNA3*, Ephrin-A3; *SIRT1*, sirtuin 1; LV, left ventricular.

## 4. The Function of Exercise-Induced miRNA in Cardiometabolic Diseases

Several risk factors, including abdominal obesity, high blood pressure, dyslipidemia, elevated triglycerides (TG), low HDL cholesterol and elevated fasting blood sugar, lead to oxidative stress, systemic inflammation, myocardial lipotoxicity, disturbed energy homeostasis, coronary endothelial dysfunction, as well as left ventricular remodeling and dysfunction. These, in turn, result in a spectrum of cardiometabolic diseases, such as hypertension, insulin resistance, diabetes, and non-alcoholic fatty liver disease, representing some of the most serious health challenges of the 21st century [63, 64]. Although medications for the treatment of cardiometabolic diseases have made significant advances, the risk of HF in patients with cardiometabolic diseases does not decline. Interestingly, a sustainable intensity of exercise has emerged as an effective synergistic therapy to mitigate and combat adverse alterations that impair cardiovascular function. Further, this can regulate miRNA levels, which have emerged as key molecular modulators of beneficial adaption and pathophysiological stresses [65]. Dysregulation of miRNAs occurs in multiple pathologic processes that regulate cell apoptosis and other cellular functions leading to cardiometabolic diseases, including diabetic cardiomyopathy [45]. The protective effects of exercise on the coronary arteries and heart during the onset and progression of diabetic heart disease (DHD) are found to be mediated by the normalization of cardiovascular-enriched miRNAs [66, 67]. Several studies have shown that exercise training (ET) could upregulate miRNA-126-3p levels and is cardio-protective [61, 68, 69, 70, 71]. A significant reduction of miR-126-3p expression was associated with the downregulation of RAF1 (raf-1 proto-oncogene, serine/threonine kinase), vascular endothelial growth factor (VEGF), and phosphoinositide 3-kinase (PI3K), high blood glucose level, insulin resistance, and angiogenesis impairment in diabetes, all of which could be reversed by exercise. This suggests that miRNA-126-3p could be a valuable therapeutic strategy against diabetes [70]. Another study found that in Wistar rats with diabetes, miR-126-3p and angiogenesis were reduced, while miR-210-3p was increased. These adverse events could be reversed by garlic treatment or long-term voluntary exercise. miR-126-3p and miR-210-3p correction mediated by exercise could be important for improving these functions [61]. Consistently, miR-126-3p was found to be downregulated in castrated streptozotocin-induced type I diabetes rats. Testosterone and ET could both enhance miR-126-3p expression and positively improve diabetic cardiomyopathy [71]. Many pathological changes were found in the cardiac tissue of diabetic ovariectomized rats, including increased cholesterol, triglyceride, low-density lipoprotein (LDL), decreased HDL, upregulated cell-apoptosis related proteins such as B-cell lymphoma 2 (Bcl-2)-associated X protein (BAX), CASP3, CASP8, and decreased miR-133a-5p. Exercise could promote miR-133a-5p expression, reverse nearly all the above deleterious alterations and improve the adverse effects of estrogen deficiency and diabetes. Moreover, overexpression of miR-133a-5p could reduce the extracellular matrix protein, significantly reducing cardiac fibrosis and the heart injury caused by diabetes [45, 72]. Obesity occurs owing to excessive fat accumulation in the body, which is now a global epidemic, and the incidence rate is still rising yearly. Obesity is closely associated with multiple cardiovascular and skeletal muscle diseases and could be used as an important risk marker. Considerable efforts have been made to elucidate obesity-related molecular pathways. Among them, miRNAs and their target genes are identified to play critical roles in regulating the obese phenotype and its associated comorbidities [73]. ET has been widely used for treating obesity and could combat aberrant metabolism and counteract weight gain. miR-208a-3p, a cardiac-specific miRNA, could regulate β myosin heavy chain (β-MHC) content and mediate systemic energy homeostasis by targeting *MED13*. miR-208a-3p was reduced, and its target *MED13 *was increased in obese Zucker rats (OZR). At the same time, exercise could correct dysregulated miRNA-208a-3p, thereby preventing weight gain and pathological cardiac hypertrophy while improving metabolic and cardiac disorders by increasing MED13 protein [60]. miR-16-5p was also found to be upregulated in OZR in another study. miR-16-5p could inhibit endothelial function and angiogenesis *in vitro* by regulating the expression of VEGF, VEGF receptor 2 (VEGFR2), and fibroblast growth factor receptor 1 (FGFR1) [73]. ET could restore miR-16-5p expression levels and induce cardiac angiogenesis in obese animals by downregulating miR-16-5p and upregulating angiogenic factors, including VEGF [56]. High-fat diet (HFD) induces pathological cardiac hypertrophy and cardiac fibrosis, reduces coronary reserve, and impairs cardiac function. The expression of miR-344g-5p and its target, 3-hydroxy-3-methylglutaryl-CoA synthase 2 (*HMGCS2*), was disturbed in the hearts of HFD mice. Swimming exercise could restore their expression and mitigate lipotoxicity and cardiac injury. Mechanistically, exercise attenuated lipid metabolic disorder and adverse cardiac remodeling by increasing miR-344g-5p expression, which inhibited HMGCS2 expression, preventing cardiomyocyte apoptosis and CASP3 cleavage [57]. Mammalian sterile 20-like kinase 1 (MST1) plays a key role in regulating the progression of diabetic cardiomyopathy (DCM). Physical exercise could reduce MST1 expression and attenuate cardiac systolic dysfunction and fibrosis. Mechanistically, miR-486-5p could be induced by exercise, in turn suppressing MST1 expression and preventing the apoptosis of high-glucose treated cardiomyocytes [74]. Matrix metalloprotease 9 (MMP9) was found to be activated and have deleterious effects that caused extracellular matrix remodeling and cardiac fibrosis in type 2 diabetes db/db mice. miR-455-5p and miR-29b-3p were upregulated by exercise in serum exosomes released from cardiomyocytes, both of which have binding sites in the 3’ region of *MMP9*. ET might bring unequivocal benefits against diabetic cardiovascular complications by reducing *MMP9* levels by upregulating miR-455-5p and miR-29b-3p [58]. miR-21a-5p downregulation was associated with the occurrence and progression of hyperlipidemia, caused lipid metabolism disorder and overexpression of target genes such as, fatty acid binding protein 7 (*FABP7*), 3-hydroxy-3-methylglutaryl-CoA reductase (HMGCR), acetyl-CoA acetyltransferase 1 (*ACAT1*), and oxidized low-density lipoprotein receptor 1 (*OLR1*). While aerobic exercise could normalize miR-21a-5p, mitigate lipid metabolism dysregulation, and improve hyperlipidemia [43]. Hyperglycemia in diabetes-induced endothelial dysfunction and miRNA dysregulation contributes to the development of diabetes-associated comorbidity. miR-181b was found to be reduced in the renal arteries of diabetic patients and non-diabetic patients treated with advanced glycation end products (AGEs). In addition, studies have found that miR-181b is an anti-inflammatory mediator against atherosclerosis in vasculature. Regular exercise could promote miR-181b-5p expression through 5’ adenosine monophosphate-activated protein kinase (AMPK) activation and reduce endothelial dysfunction, vascular inflammation, and oxidative stress in diabetic mice [59]. In conclusion, miRNAs have emerged as essential regulators of cardiometabolic complications and could be used for developing novel therapeutic strategies for numerous diseases.

## 5. The Function of Exercise-Induced miRNAs in Myocardial Infarction

Myocardial infarction (MI) is the most common medical emergency among cardiovascular diseases, with high morbidity and mortality. With the shift in diet structure and increase in aging population, the prevalence of MI is consistently rising, with an increasingly younger population suffering from MI. Numerous studies have suggested that exercise provides direct endogenous benefits against MI and could be used as a necessary adjuvant therapy against cardiac dysfunction post-MI. Using left anterior descending (LAD) ligation-induced MI combined with ET rat models, miRNA-497-5p expression was found to be enhanced by MI. ET could reduce miRNA-497-5p expression under physiological and under MI pathological conditions. A miRNA-497-5p antagomir (inhibitor) could mimic the benefits of exercise on MI, including reduced infarct size and improved cardiac function, whereas miRNA-497-5p agomir aggravated the infarct size post-MI and abrogated the positive effects of ET maybe through its target chloride voltage-gated channel 3 (*CLCN3*) [47]. Through a plasma miRNA profiling assay, miR-1192 was found to be increased by a four-week swim training. miR-1192 overexpression provided significant beneficial effects against hypoxia in cultured neonatal rat cardiomyocytes (NRCMs) by targeting *CASP3*. Moreover, intramyocardially injection of miR-1192 agomir could mimic the positive effects, while using its antagomir blocked the beneficial effects of exercise against MI [46]. Studies showed that exercise stress induced by a 4-week ET MI rat model could trigger hypoxia-inducible factor-1α (HIF-1α) expression that triggered miR-126-3p expression, which in turn downregulated phosphoinositide-3-kinase regulatory subunit 2 (PIK3R2) and sprouty related evh1 domain containing 1 (SPRED1). HIF-1α/miR-126-3p axis was involved in ET-induced myocardial angiogenesis post-MI by regulating PI3K/protein kinase B (AKT)/endothelial nitric oxide synthase (eNOS) and mitogen-activated protein kinase (MAPK) signaling pathways, thereby improving cardiac function against myocardial injury [68]. In the same year, another study also showed that miR-126-3p was upregulated by exercise, and the expression of its target gene, *SPRED1*, was downregulated. ET, as well as the administration of soluble epoxide hydrolase inhibitor (sEHi) - 1-Trifluoromethoxyphenyl-3-(1-propionylpiperidin-4-yl) urea (TPPU), could increase the levels of epoxyeicosatrienoic acids (EET) and exert positive effects on the angiogenic function of endothelial progenitor cells (EPCs) to improve cardiac function post-MI. miRNA-126-3p overexpression induced by TPPU could be partially mediated by extracellular signal-regulated kinase (ERK) and p38 MAPK phosphorylation and inhibit SPRED1 under exercise conditions in mice to protect against myocardial injury [69]. MI-induced downregulation of miR-1 and upregulation of miR-214-3p regulated their respective targets, sodium/calcium exchanger 1 (*NCX*) and sarcoplasmic reticulum calcium ATPase-2a (*SERCA2A*), accordingly. ET could restore the expression of miR-1-5p and miR-214-3p to baseline, thus leading to the normalization of ${\mathrm{Ca}}^{2+}$ handling, left ventricular (LV) compliance in infarcted hearts, and restoring ventricular function [44]. Increased collagen deposition and cell necrosis are found in cardiac tissue after MI, which results in reduced ventricular compliance and cardiac dysfunction. Swimming training can upregulate cardiac miR-29 family members, miR-29a, and miR-29c, but not miR-29b. These, in turn, reduce the expression of collagen I and collagen III in the border region (BR) and remote myocardium (RM), thereby improving cardiac function after MI [49]. The function of miR-29a-3p in mediating cardiac fibrosis post-MI under exercise conditions has been elucidated in another study. Controlled intermittent aerobic exercise was found to reduce fibrosis and inhibit the TGF-β pathway by upregulating the expression of miR-29a-3p and miR-101a-3p. These microRNAs then target the mRNAs encoding collagen and other proteins involved in fibrosis, resulting in reduced fibrosis and scar formation in cardiac tissue after MI [48]. One of the major features of HF is the loss of cardiomyocytes and failed endogenous cardiomyocyte generation. The adult heart exhibits a minimal capacity for cardiomyogenesis, and the molecular mechanism is still unclear. Newer mononucleate or diploid cardiomyocytes could be labeled with the incorporation of ^15^N-thymidine and detected by multi-isotope imaging mass spectrometry (MIMS). 8-week running exercise significantly increased the production of new cardiomyocytes in adult mice. Furthermore, ET induced robust endogenous cardiomyocyte generation in an extended border zone of the infarcted area after myocardial infarction. miR-222-3p was observed to be upregulated by ET in both animal models and humans, and its inhibition completely abrogated cardiomyogenesis stimulated by exercise. This suggests that miR-222-3p is an essential regulator of cardiomyogenic exercise response in both normal and injured adult mouse hearts and contributed to the benefits of exercise [75]. Aerobic exercise and statins could induce the expression of miR-146a-5p and miR-126-3p and reduce miR-155-5p. Moreover, miRNA-146a-5p binds to the 3’ untranslated region of the* TRAF6* gene and inhibit its expression, ultimately reducing vascular TRAF and TLR4 signaling and vascular inflammatory response in atherosclerosis [51]. Exercise can induce the increase of miR-20a-5p, which interacts with $3^{\prime }$UTR of PTEN to downregulate its expression, promoting cell survival and proliferation through the activation of PI3K/Akt signaling pathway, reducing the incidence of coronary artery disease [50]. These findings demonstrate the cardioprotective effects against MI by exercise mediated by miRNA and its target networks. Furthermore, these exercise-miRNAs could be novel therapeutic targets in treating MI.

## 6. The Function of Exercise-Induced miRNAs in Ischemia-Reperfusion Injury (IRI)

Reperfusion therapy, including thrombolytic and fibrinolytic drugs, percutaneous coronary intervention (PCI), and coronary artery bypass grafting surgery, are the most common treatments for MI in clinical practice that greatly reduce acute death. However, restoring blood flow to ischemic cardiac tissue will inevitably lead to myocardial injury, pathological ventricular remodeling, and even cause chronic heart failure (CHF) and death. Effective ischemia-reperfusion injury (IRI) treatment is still an urgent requirement in current clinical practice. Accumulating evidence has shown that exercise-induced cardiac adaption can resist cardiac dysfunction caused by IRI, and miRNAs are essential during this process.

To identify miRNAs that mediate exercise-induced physiological cardiac growth, two ET models, a voluntary wheel running and a ramp swimming or sedentary control, were applied to mice for three weeks. miR-222-3p was found to be upregulated in both ET models. Overexpression of miR-222-3p could enhance cardiomyocyte growth and proliferation by targeting* KIP1* (*P27*), homeodomain interacting protein kinase 1 (*HIPK1*), *HIPK2*, and homeobox-containing 1 (*HMBOX1*). miR-222-3p inhibition could enhance serum deprivation and doxorubicin (DOX)-induced cardiomyocyte apoptosis, while the converse was observed upon miR-222-3p overexpression. Functional rescue experiment by miR-222-3p inhibition showed that it was necessary for exercise-induced cardiac growth. Moreover, miR-222-3p overexpression significantly attenuated pathological cardiac remodeling and cardiac dysfunction after IRI [55]. Using the three-week swimming or voluntary wheel exercise model, miR-17-3p was also identified to be significantly upregulated by ET, specifically in heart tissue. miR-17-3p overexpression could promote cardiomyocyte growth and proliferation, and protect against oxygen-glucose deprivation/reperfusion (OGDR)-induced cardiomyocyte apoptosis by directly targeting *TIMP-3 *and indirectly inhibiting* PTEN*. Inhibition of miR-17-3p can attenuate exercise-induced cardiac growth *in vivo*. In addition, mice administrated with miR-17-3p agomir (mimic) could be protected from adverse cardiac remodeling after myocardial IRI [54]. miR-486-5p was previously found to be upregulated in skeletal muscle and heart in ET [55, 74, 76]. Most recently, miR-486 was found to be significantly downregulated in the myocardial tissue post-IRI. In addition, miR-486-5p overexpression reduced OGDR-driven cardiomyocyte apoptosis. It protects mice against cardiac dysfunction and myocardial apoptosis post-IRI by targeting* PTEN* and *FOXO1* and activating AKT/mammalian target of rapamycin (mTOR) signaling. Depletion of miR-486 abrogated the ET’s protective effects, suggesting it is necessary for cardio-protection [77]. Collectively, these results showed that miR-222-3p, miR-17-3p and miR-486-5p are key participants in exercise-induced physiological cardiac adaption, and play critical roles in protecting against adverse cardiac stress and dysfunction.

It is not only the exercise-induced miRNAs from heart tissues that mediate protective effects for cardiac injury. Circulating exosome-delivering miRNAs in serum have also been found to be essential for exercise-induced cardioprotection [78, 79]. Circulating exosomes isolated from the plasma of 4-week swimming exercise-trained rats provided remarkable beneficial effects, especially against IRI. miRNA sequencing combined with quantitative reverse transcription polymerase chain reaction (qRT-PCR) verification identified 12 differentially expressed exosome-derived miRNAs in exercise-induced rats. Functional analysis showed that miR-342-5p is necessary for reducing hypoxia/reoxygenation-induced cardiomyocytes apoptosis, attenuating cardiac dysfunction and myocardial injury by targeting *Caspase 9* (*CASP9*) and *JNK2*. This enhances the survival signaling through the phosphorylation of Akt (p-Akt) by targeting phosphatase gene, *PPM1F*, in cardiac tissue after IRI [53]. Exercise could induce the expansion of brown adipose tissue (BAT), the thermogenic tissue in mice, and surgical BAT ablation could reduce the protective effects of exercise against IRI. The small extracellular vesicles (sEV) derived from BAT have been shown to communicate with the heart, regulate cardiomyocyte survival and mediate exercise-induced cardioprotection against myocardial IRI. BAT miRNAs, including miR-125b-5p, miR-128-3p, and miR-30d-5p, are involved in this process by targeting a series of molecules, such as *MAP3K5*, *MAP2K7*, and *MAP2K4* to suppress the proapoptotic MAPK pathway [52]. Inhibition of the miRNAs in BAT specifically abrogated their increase in plasma sEVs and hearts of exercise-trained mice and consequently reduced the beneficial effects of ET.

Exosome-miRNAs are also found to regulate interorgan crosstalk. Cardiac dysfunction induced by particulate matter 2.5 (PM2.5) was found to be associated with miR-421-3p in sEV released from the injured lung. This was transferred to cardiac tissue and exerted its function by regulating its target gene, *ACE2*, causing myocardial cell apoptosis and myocardial injury. Cardiac injury caused by PM2.5 resulted from crosstalk between the lungs and heart and was secondary to lung injury. Inhibition of miR-421-3p could significantly attenuate cardiac dysfunction induced by PM2.5 in mice [80]. Cardiac homing peptide (CHP)-linked plasma circulating extracellular vesicles (EVs) delivered either by percutaneous intracoronary delivery (in a canine model) and myocardial injection just before reperfusion (in murine model), possibly mediated by miR-486-5p, could protect the heart against IRI. Moreover, the depletion of miR-486-5p in EVs abrogated the protective roles of circulating EVs on IRI, suggesting that EV-miR-486 is crucial for the cardioprotective effect [81]. Taken together, these suggest that miRNAs originating from heart tissue or those delivered by EVs derived from remote organs are essential for mediating cardioprotection.

## 7. The Function of Exercise-Induced miRNA in Cardiac Remodelin*g*

Cardiac remodeling is well recognized as the primary pathological basis of multiple cardiovascular diseases. Pathological cardiac remodeling occurs in response to numerous stresses wherein the initial aim is to maintain cardiac function despite various stress conditions. Sustainable stress conditions would induce cardiac remodeling transition from the adaptive stage to maladaptive alterations, leading to cardiac dysfunction and HF. Mounting studies have shown that miRNAs are essential in regulating cardiac remodeling by controlling the target gene expression. Obesity is considered a low-level systemic inflammation state that contributes to the development of atherosclerosis, insulin resistance, hypertension, endothelial dysfunction, and cardiometabolic diseases, all of which are associated with pathological cardiac remodeling. miR-1-5p and miR-29c-3p were observed to regulate obesity-related cardiac remodeling by targeting the sodium/calcium exchanger NCX1 and collagen expression, respectively. While miRNA-29c-3p was decreased, collagen expression was increased, whereas when miR-1-5p was upregulated, the expression of its target gene, calcium signaling protein NCX1, was decreased in OZR. Aerobic ET could restore all these parameters, consequently attenuating cardiac dysfunction and protecting the heart from an abnormal increase in extracellular matrix components and pathological cardiac hypertrophy by regulating miRNAs [82].

Exercise could promote the dedifferentiation and proliferation of original cardiomyocytes and produce new cardiomyocytes by regulating various cytokines, transcription factors, and miRNAs. Some miRNAs involved in cardiomyocyte regeneration or proliferation have been found to promote heart repair after myocardial injury [83, 84, 85]. Beneficial cardiac remodeling against deleterious stressors also occurred in response to exercise-induced adaptions that cause significant changes in myocardial structure and function [83, 86]. Robust evidence has shown that exercise could upregulate cardiac miR-222-3p and miR17-3p, which in turn could induce significant cardiac hypertrophy [54, 55].

Several circulating miRNAs in serum are found to be altered by exercise, exhibiting numerous cardiac biological, and physiological effects mediating structural and functional adaptions. A total of thirty-six circulating cardiac-related miRNAs were investigated in a study. Most miRNAs upregulated by acute exercise and returned to normal for extended periods. Five of them (miR-1-5p, miR-133a-5p, miR-146a-5p, miR-206-3p, and miR-221-3p) were found to be directly related to cardiac adaptation parameters. In contrast, two of them (miR-1-5p and miR-133a-5p) were associated with cardiac hypertrophy, suggesting that circulating microRNAs could act as promising biomarkers for evaluating the effects of exercise on cardiac hypertrophy and exercise-induced cardiac adaptation [87]. Bioinformatics databases, including MirPath v.3, TargetScan, Kyoto Encyclopedia of Genes and Genomes (KEGG), and Gene Ontology (GO) analyses, were applied to analyze twenty-three exercise-regulated microRNAs from eight published studies to identify functional annotations and potential pathways associated with physiological cardiac hypertrophy induced by exercise. Various miRNA targets and biological pathways most likely associated with exercise-induced physiologic cardiac hypertrophy were identified, such as arrhythmogenic right ventricular cardiomyopathy (ARVC), fatty acid elongation, and extracellular matrix (ECM)-receptor interaction [88].

## 8. The Function of Exercise-Induced miRNAs in Heart Failure

Cardiovascular diseases (CVD) are the leading cause of death in developed and developing countries accounting for more than one-third of deaths globally. HF is a common end phase in many CVDs and is one of the fastest-growing global health problems [89]. There is still a lack of effective treatment that could reverse HF in clinical setting. Studies on HF patients and HF animal models showed that ET has cardio-protective effects, suggesting that ET could be used to excavate targets for developing novel therapeutic strategies. miRNA expression patterns are substantially altered following exercise stress due to changes in transcription, post-transcriptional regulation, and miRNA biogenesis control, and could reveal the cardio-protective mechanisms of ET [90]. The cause-effect relationship between miRNA regulation and HF and the potency of ET in reversing miRNAs toward baseline levels were investigated in a study using an exercise-trained HF model in rats. HF caused the dysregulation of 55 miRNAs; 18 were restored to normal levels by high-intensity endurance training, thus, contributing to the benefits of ET in improving cardiac function by bettering ${\mathrm{Ca}}^{2+}$ cycling and reducing arrhythmias [91]. Of the different exercise-miRNAs identified in this study, miR-31a-5p and miRNA-214-3p showed the most promising beneficial effects against HF-cardiac phenotype demonstrated by overexpression studies on human induced pluripotent stem cell-derived cardiomyocytes (hiPSC-CMs). Another study used ascending aortic stenosis (AS)-induced HF rat model to explore the cardioprotective mechanisms of ET in attenuating cardiac abnormalities. A set of 14 dysregulated miRNAs that responded robustly to ET in HF were used to construct miRNA-mRNA regulatory networks. These were observed to be involved in regulating programmed cell death, TGF-β signaling, cellular metabolic processes, cytokine signaling, and cell morphogenesis [92]. The alteration of miRNAs by pathologic stress and the restoration by ET was observed in patients with HF. Genome-wide serum miRNA expression profiling analysis of hospitalized patients with HF and volunteer control also showed that certain dysregulated circulating miRNAs in HF could be restored to nonpathological levels by exercise-based cardiac rehabilitation (CR). This indicates that the beneficial effects of CR for HF may result from multiple mechanisms that involve the regulation of miRNAs and their targets. The expression of two miRNAs was significantly different in HF before and after CR. Hsa-miR-125b-3p was significantly downregulated, while hsa-miR-1290 was significantly upregulated in patients before CR [93]. Serum circulating miR-21-5p, miR-378-3p, and miR-940 levels were also found to be significantly upregulated in response to an acute exhaustive exercise in patients with congestive HF. However, these miRNAs’ association with congestive HF must be further investigated [94]. The function of HDL in stimulating nitric oxide (NO) generation by endothelial cells (ECs) was observed to be impaired in patients with CHF involving HDL-induced miRNAs. Comparative analysis of selected miRNA expression levels in CHF patients and healthy subjects found that the expression of pro-angiogenic miRNAs, such as miR-126 and miR-21, positively regulated by HDL, were reduced in CHF and could be reversed by ET. This could contribute to the beneficial roles of ET, specifically in hindering atherogenesis and endothelial dysfunction in CHF [42].

## 9. Medications Targeting miRNAs in Treating Cardiovascular Diseases

miRNAs could act as novel therapeutic targets in treating human diseases, particularly cardiovascular diseases. Based on preclinical studies, various strategies are being developed to modulate miRNA activity to treat heart diseases [95]. miR-210-3p has been reported to regulate angiogenesis after renal IRI by targeting the VEGF signaling pathway. Huoxue-Anxin Recpe (HAR), a novel Chinese Traditional Herb Medicine formulation, could upregulate the expression of miR-210-3p and VEGF, thereby reducing the infarct size, alleviating interstitial fibrosis and improving cardiac function in rats with acute MI [96]. Studies have shown that miR-132-3p can be used as a therapeutic target for HF, and a preclinical trial demonstrated the therapeutic efficacy of anti-miR-132-3p in various models [97]. Inhibition of miR-92a-3p has been shown to exhibit several beneficial effects towards cardiovascular diseases. Inhibiting miR-92a-3p with antisense molecule can improve vascularization after myocardial infarction and blood circulation after posterior limb ischemia. In addition, inhibition of miR-92a-3p could accelerate wound healing in animal models with and without metabolic syndrome. MRG-110 is a locked nucleic acid-based antisense oligonucleotide targeting miR-92a-3p and has been shown to have therapeutic effects on cardiovascular disease as well as wound healing in a human study [98]. The expression level of miR-1-5p was significantly upregulated in $H_{2}$$O_{2}$-treated cardiomyocytes and transgenic (TG) mice post-MI. Metformin (Met) was found to improve the conduction delay of the heart by inhibiting the expression of miR-1-5p [99]. Astragaloside IV (ASG/AS-IV), a cycloartane-type triterpene glycoside chemical, which is one of the active ingredients of Astragalus extract (AE), could increase the downregulated miR-135a-5p in the fibrosis model and inhibit its target gene *TRPM7*. In addition, positive feedback exists between the increase of TRPM7 and activation of the TGF-β/Smads pathway, which all contribute to the development of cardiac fibrosis. Therefore, ASG and AE could inhibit cardiac fibrosis by regulating the miR-135a-5p -TRPM7-TGF-β/Smds pathway [100]. In addition, ASG could inhibit the expression of miR-1-5p in cardiac tissues and regulate cardiomyocyte apoptosis by targeting *BCL-2*. Furthermore, both ASG and miR-1-5p inhibitors could improve cardiac insufficiency by regulating calcium and mitochondria-related proteins [101]. DOX significantly upregulated the expression of miR-140-5p in H9C2 cells and heart tissues of rats and mice. In accordance, diosgenin could downregulate miR-140-5p alleviating the myocardial oxidative stress induced by DOX. This miRNA regulates nuclear factor erythroid 2-related factor 2 (NRF2) and sirtuin 2 (SIRT2) signaling pathways, affecting the expression of heme oxygenase-1 (HO-1), The nicotinamide adenine dinucleotide phosphate (NAD(P)H) quinone oxidoreductase 1 (NQO1), glutamate-cysteine ligase modifier Subunit (GCLM), kelch-like ECH associated protein 1 (KEAP1) and forkhead transcription factor O subfamily member 3a (FOXO3a) [102]. Hyperin has been shown to upregulate miR-138-5p, which can inhibit the expression of mixed lineage kinase 3 (MLK3) and the phosphorylation of its downstream signaling targets. In addition, lipocalin-2 (LCN2) is also inhibited as a target of miR-138-5p. Therefore, Hyperin can promote cardiomyocyte survival, reduce hypoxia-induced apoptosis, and play a cardioprotective role by regulating miRNA-138-5p [103]. The antagomir-132-3p injection, by inhibiting its target miR-132-3p, can improve FOXO3 protein levels and attenuate calcineurin/nuclear factor of activated T cells (NFAT) signal transduction induced by pressure overload, thereby abrogating cardiac hypertrophy and HF [104].

## 10. Targeting miRNAs in Clinical Trials for Treating Cardiac Diseases

Currently, many drug discovery programs focus on the development of miRNA-based therapies. In one such program, antisense oligonucleotide miravirsen, the first miRNA-targeting drug, modified by locked nucleic acid (LNA) antisense oligonucleotides, has been used to target miRNA-122 for the treatment of hepatitis C in the liver [105].

Many miRNA-targeting drugs are being investigated in clinical trials. Cardiac microRNA-132-3p (miR-132) levels are elevated in patients with HF, and CDR132L, a specific antisense oligonucleotide that inhibits miR-132-3p, is being used to treat patients with HF. CDR132L improved cardiac function compared to the placebo as well as being safe and well tolerated without significant dose-limiting toxicity [106]. Rosuvastatin treatment significantly reduced the incidence of cardiovascular events in patients with acute coronary syndromes undergoing PCI, compared with placebo, possibly by inhibiting miR-155-5p/Src homology 2-containing inositol phosphatase-1 (SHIP-1) signaling [107]. In addition, another study found that higher miR-33b-5p was associated with lower ATP-binding cassette transporter (ABCA)1 mRNA in hypercholesterolemic patients, and rosuvastatin administration may revert this condition. Mechanistically, Rosuvastatin could down-regulate the miR-33b-5p and reverse the lower expression of ABCA1, playing a role in the treatment of atherosclerosis [108]. In patients undergoing noncoronary artery cardiac surgery, a randomized controlled trial showed that simvastatin treatment significantly reduced miR-15a-5p levels, resulting in increased expression of its target gene *BCL-2* in cardiomyocytes. This inhibited myocardial apoptosis and protected the myocardium, as demonstrated in study [109]. Visceral obesity can cause various cardiovascular diseases. Evidenced from the Cardiovascular Effects of Chronic Sildenafil in Men with Type 2 Diabetes (CECSID) trial demonstrated that sildenafil, a phosphodiesterase-5 (PDE5) inhibitor, decreased the expression of miR-22-3p compared to the placebo, and sirtuin1 (SIRT1), a target of miR-22-3p, was upregulated, leading to a shift in adipose tissue cell composition towards a less inflammatory status [110]. Robust evidence has elucidated that Propofol can ameliorate IRI in patients by increasing the expression of miR-30b-5p and targeting Beclin-1, thereby inhibiting excessive autophagy and apoptosis [111]. Several studies showed thatmiR-126-3p is important for maintaining vascular integrity [21, 112, 113, 114]. miR-126 deficiency was found to impair the differentiation and diversification of embryonic ECs, suggesting its essential role in maintaining EC heterogeneity [112]. miR-126-5p could maintain the integrity of endothelial cells under high shear stress and autophagy by exerting non-canonical posttranslational functions. The binding of miR-126-5p to argonaute-2 (AGO2) results in the formation of a complex with MEX3A, which takes place on the surface of autophagic vesicles. This complex subsequently enters the nucleus. Once inside, miR-126-5p dissociates from AGO2 and interacts with CASP3 to suppress caspase dimerization and inhibit its activity, thereby limiting cell apoptosis [21]. The potential roles of miR-126-3p in resisting atherosclerosis were verified in human umbilical vein endothelial cells (HUVECs). miR-126-3p was significantly reduced in the HUVECs treated with ox-LDL. Whereas miR-126-3p mimics could restore the autophagic flux by inhibiting PI3K/Akt/mTOR signaling pathway and reducing the ECs injury induced by ox-LDL [113]. Another study found that miR-126-5p is expressed in endothelial cells and retinal ganglion cells (RGC) of the postnatal retina and is involved in protecting endothelial cells from apoptosis by regulating SET domain containing 5 (SETD5) during the establishment of retinal vascular system [114]. miR-27a-3p can increase the expression of angiotensin-converting enzyme (ACE), which leads to cardiovascular inflammation and remodeling by the nuclear factor kappa-light-chain-enhancer of activated B cells (NF-κB) pathway activation, ultimately leading to hypertension. Mitoquinone mesylate (MitoQ) is a supplement that acts on mitochondria and weakens reactive oxygen species (ROS). Studies have shown that the combination of endurance training and MitoQ, compared with either MitoQ administration or endurance training alone, could significantly increase miR-126-3p and reduce miR-27a-3p levels, thus improving the production of mitochondrial ROS and alleviating cardiac function in patients with hypertension [115]. A randomized clinical study showed that atorvastatin could significantly reduce the level of miR-34a-5p and affect the expression of its target SIRT1 in EPCs. Thus, atorvastatin could increase SIRT1 and improve endothelial function in coronary artery disease [62]. The CENTRAL trial demonstrated that lifestyle interventions reduced the expression of circulating miR-99-5p/100-5p, consequently improving body fat distribution, reducing fat depots, and abrogating cardiac dysfunction in diabetes [116]. The dysregulation of mir-146a/b-5p expression may lead to the long-term activation of TLR4 and its downstream signaling in peripheral blood mononuclear cells (PBMC) of patients with coronary heart disease. At the same time, a clinical study showed that the combination therapy of statins (atorvastatin) and telmisartan (angiotensin II receptor blocker, ARB) reduced both miR-146a/b-5p and Toll-like receptor 4 (TLR4) signaling in patients with coronary artery disease. miR-146a/b-5p can regulate TLR4 downstream molecules interleukin-1 receptor-associated kinase 1 (IRAK1) and tumor necrosis factor receptor-associated factor 6 (TRAF6), thereby resisting atherosclerosis [117]. A clinical study has shown that EPOC training can upregulate the expression of miR-20a-5p and downregulate the expression of miR-125b-5p. miR-20a-5p and miR-125b-5p have been found to be involved in the improvement of hypertension using high-intensity interval training [118]. miR-132-3p can be used as a therapeutic target for HF as demonstrated by a preclinical trial using an antimiR-132-3p treatment regimen, which shows a high clinical potential with excellent pharmacokinetics, safety, tolerability, and dose-dependent pharmacokinetics/pharmacodynamics (PK/PD) relationship [97]. In conclusion, anti-miRNA drugs designed specifically based on the chemical structure of miRNA, including antisense oligonucleotides (antimiRs, blockmiRs), miRNA sponges, miRNA mimics, and miRNA mowers, or pharmaceutical drugs that could regulate miRNA expression, have potential to be effective therapeutic strategies in treating cardiovascular diseases [95].

## 11. Conclusions

It has been nearly 30 years since the discovery of these small non-coding single-stranded nucleic acids called miRNAs. These act as post-transcriptional gene regulators with a critical role in nearly all biological processes, including exercise-induced cardio-protection. Exercise-miRNAs are essential components for regulating the positive effects against pathological alterations. Furthermore, miRNA-drug therapies could mimic the beneficial effects of exercise and have a promising role in patients where exercise therapy is not an option. Using miRNAs as drug targets may aid in treating diseases with no viable therapeutic options, such as undruggable proteins. These can now be targeted and corrected through their upstream miRNA gene regulators. However, one of the main limitations is the chemical structure of miRNAs. Therefore, generating potential drug molecules with the necessary pharmacokinetic properties is still challenging and require careful optimization in the drug discovery process.
